# Electroconvulsive therapy use in New South Wales between 1944 and 1949

**DOI:** 10.1177/10398562241306899

**Published:** 2024-12-12

**Authors:** Brian Draper, Chanaka Wijeratne

**Affiliations:** Discipline of Psychiatry and Mental Health, 7800 University of NSW, Sydney, AU -NSW, Australia

**Keywords:** ECT, history, outcomes

## Abstract

**Aims:**

To identify clinical and other factors associated with the use of electroconvulsive therapy (ECT) in New South Wales for the period 1944–1949 and to compare with contemporaneous practice.

**Method:**

Annual reports of the Inspector-General of Mental Hospitals in NSW (1944/45 to 1948/49) were examined.

**Main findings:**

Seven hospitals reported a total of 8964 courses of treatment during the period. Demographically, 60.6% of patients who received ECT were female, and rural hospitals reported 13.6% of courses. ECT was as likely to be given for non-affective psychoses (37.7%), as for affective psychoses (35.0%). ECT was also administered for confusional states and non-psychotic disorders. Better outcomes were observed for depression, anxiety and confusional states than non-affective psychoses. Recovery rates declined over the study period. Mortality was <1%.

**Conclusions:**

The clinical indications for ECT in the mid-twentieth century in NSW were much broader than currently. There has been no change in the sex ratio of patients administered ECT or reported mortality.

Although the origins of electroconvulsive therapy (ECT) can be traced back to the eighteenth century,^1^ it was the work of Cerletti and Bini in Italy in April 1938 that heralded modern ECT.^2^ This occurred in the context of there being no effective psychotropic drugs, with alternative treatments for severe psychoses being insulin coma therapy, pentamethylenetetrazol (Cardiazol) convulsion therapy, or psychosurgery, each of which had significant adverse effects.^2^ Ladislaus von Meduna, who pioneered intravenous Cardiazol convulsion therapy to treat schizophrenia in 1934, had initially used intramuscular camphor which he abandoned due to its inconsistent composition and speed of action.^3^ By 1940, ECT was being used to treat a wide range of severe mental disorders in North America and Europe,^2^ sometimes with remarkable results as noted in newspaper reports.^4^ ECT was regarded as being particularly effective in the treatment of psychoses and during the 1940s this was further narrowed to the affective psychoses, although it was also used more controversially in a range of other conditions including neuroses, personality disorders and anorexia nervosa.^5–7^

Insulin and Cardiazol were introduced in New South Wales (NSW) in 1937–38 for the treatment of schizophrenia. The NSW Inspector-General of Mental Hospitals noted in his annual report in 1939 that insulin treatment was limited by the need for additional staff and Cardiazol was showing promising results.^8^ Indeed, Dr S Evan Jones examined the results of Cardiazol therapy for the period 1937 to 1941 and found that 61% of manic depressive states and 29% of schizophrenia cases recovered.^9^ By 1940 stocks of Cardiazol in NSW were exhausted, and although Phrenazol was being used in its place with good results in schizophrenia and other psychoses, this proved the precipitant to NSW procuring two ECT machines to replace or partially replace Cardiazol.^10^

The first ECT machine in Australia was built by Dr Hugh Birch at Parkside Hospital, Adelaide in 1941.^11^ In NSW, the first ECT machines were imported from England in July 1942 and used at Callan Park Hospital, with early success reported in the newspapers in October 1942.^12,13^ Australian research into the use of ECT first appeared in 1948 when Bostock and Phillips from Brisbane published an overview of the use of ECT in the Medical Journal of Australia, including the outcomes of the treatment of 100 cases of anxiety and depression from the Brisbane General Hospital.^14^ There is no other published Australian research that examined ECT use and its outcomes in the 1940s.

In this study, the statewide use of ECT in NSW in the period 1944–1949 is examined from information published in the annual reports of the Inspector-General of Mental Hospitals in NSW. The aims are to describe the mental disorders for which ECT was used in this period, the treatment outcomes obtained for each disorder, sex differences in the use of ECT, and whether the use of ECT, and the outcomes obtained, changed over the period of the study and compared with contemporary practice.

## Methods

The Inspector-General of Mental Hospitals in NSW, under the requirements of the Lunacy and Inebriates Act 1937, presented an Annual Report to the NSW Parliament with details of the provision of mental health services in NSW. The reports are available in the State Library of NSW. Between 1945 and 1949, these reports included tables with non-identifiable details of the provision of ECT in the previous financial year, including the hospitals where it was used, the mental disorders it was used for, the sex of the patient and the treatment outcomes. There was no information about patient age, the number of ECT treatments administered in a course, the specific characteristics of ECT, the manner in which consent was obtained, or socio-economic factors.^15–19^

Patient sex, the frequency and outcomes of treatment (recovered, relieved, not improved, died) of ECT use in each mental disorder were determined. Comparison of treatment effectiveness was calculated by the proportions in each outcome category for each disorder. Comparison of the use of ECT in each hospital was made regarding the types of mental disorders treated. Treatment use and outcomes over time were examined.

Ethical approval was obtained from the University of NSW Human Research Ethics Committee (iRECS7225).

## Results

In the five financial years covered in these reports, there were seven hospitals reporting their use of ECT – Broughton Hall (5 years), Orange (5 years), Callan Park (4 years), Gladesville (4 years), Morisset (4 years), Parramatta (4 years), and Kenmore (2 years). Most of the treatment courses were undertaken at Broughton Hall (*n* = 4590, 51.2%), followed by Callan Park (*n* = 1214, 13.5%), Gladesville (*n* = 1152, 12.9%), Parramatta (*n* = 806, 9.0%), Morisset (*n* = 690, 7.7%), Orange (*n* = 357, 4.0%) and Kenmore (*n* = 167, 1.9%). The combined data for the five financial years is presented in Table 1. ECT was as likely to be given for non-affective psychoses (schizophrenia and paraphrenia) (37.7%) as for affective psychoses (manic depressive psychosis – depression, mania and mixed) (35.0%).

**Table 1.** Outcomes of electroconvulsive therapy in NSW 1944/45–1948/49*.

Mental disorders**	Treatments *n*	Female *n* (%)	Recovered *n* (%)	Relieved *n* (%)	Not improved *n* (%)	Died *n* (%)
MDP*** – Manic states	693	477 (68.8%)	358 (51.7%)	206 (29.7%)	129 (18.6%)	0
MDP – Depressive states	2419	1535 (63.5%)	1709 (70.6%)	535 (22.1%)	172 (7.1%)	3 (0.1%)
MDP – Mixed states	27	18 (66.7%)	12 (44.4%)	11 (40.7%)	4 (14.8%)	0
Schizophrenia	2342	1129 (48.2%)	519 (22.2%)	1046 (44.7%)	777 (33.2%)	0
Paraphrenia	1025	702 (68.5%)	364 (35.5%)	417 (40.7%)	244 (23.8%)	0
Confusional states	503	352 (70.0%)	326 (64.8%)	121 (24.1%)	55 (10.9%)	1 (0.2%)
Demented states	139	115 (82.7%)	9 (6.5%)	40 (28.8%)	90 (64.7%)	0
Other states	12	5 (41.7%)	0	8 (66.7%)	3 (25.0%)	1 (8.3%)
Dementia paralytica	20	4 (20.0%)	3 (15.0%)	10 (50.0%)	7 (35.0%)	0
Congenital mental deficiency	78	34 (43.6%)	6 (7.7%)	30 (38.5%)	42 (53.8%)	0
Epilepsy	58	27 (46.6%)	5 (8.6%)	32 (55.2%)	21 (36.2%)	0
Hypochondria	72	61 (84.7%)	35 (48.6%)	27 (37.5%)	10 (13.9%)	0
Hysteria	281	230 (81.9%)	184 (65.5%)	65 (23.1%)	31 (11.0%)	1 (0.4%)
Anxiety states	832	477 (57.3%)	601 (72.2%)	182 (21.9%)	49 (5.9%)	0
Anxiety hysteria	198	179 (90.4%)	135 (68.2%)	51 (25.8%)	12 (6.1%)	0
Neurasthenia	127	21 (16.5%)	60 (47.2%)	50 (39.4%)	17 (13.4%)	0
Obsessional states	78	52 (66.7%)	42 (53.8%)	28 (35.9%)	7 (9.0%)	1 (1.3%)
Psychopathic states	60	17 (28.3%)	18 (30.0%)	31 (51.7%)	11 (18.3%)	0
**Total**	**8964**	**5435 (60.6%)**	**4386 (48.9%)**	**2890 (32.2%)**	**1681 (18.8%)**	**7 (0.0008%)**

*Extracted from Reports of Inspector-General of Mental Hospitals^14–18^.

**The terminology and order used for the mental disorders as appears in the reports.

***MDP – Manic Depressive Psychosis.

The use of ECT in some types of mental disorders mainly occurred at Broughton Hall – anxiety hysteria (100%), hypochondria (100%), hysteria (99.6%), anxiety states (98.3%), neurasthenia (97.6%), psychopathic states (91.7%) and epilepsy (60.3%). There were no consistent patterns of change over time in the use of ECT across the hospitals for various mental disorders. For example, the use of ECT for schizophrenia declined at Broughton Hall, Morisset and Parramatta, while it increased at Orange, Callan Park and Gladesville. In their first year of ECT use, Kenmore (37 courses) and Orange (27 courses) gave ECT for demented states with poor outcomes, then much less in later years. In contrast Gladesville had no initial use of ECT for demented states, but by 1948/49 gave 20 courses.

The outcomes of ECT declined over time. Combining all diagnostic groups, 1195 (59.2%) recovered and 425 (21.1%) were relieved in 1944/45, while 651 (34.3%) recovered and 884 (46.6%) were relieved in 1948/49. There was no change in the proportion that did not improve which was 19% in both years and the number of deaths did not change. Recoveries in specific mental disorders are presented in Table 2. At Gladesville Hospital in 1948/49 it was noted that 152 patients (49.8%) had previous courses of ECT, the only information available on this issue.

**Table 2.** Electroconvulsive therapy in NSW: Recoveries in 1944/45 compared with 1948/49*.

Mental disorders**	1944/45 treatments *n*	1944/45 recovered *n* (%)	1948/49 treatments *n*	1948/49 recovered *n* (%)
MDP*** – Manic states	146	69 (47.3%)	151	66 (43.7%)
MDP – Depressive states	491	427 (87.0%)	539	337 (62.5%)
MDP – Mixed states	5	5 (100.0%)	12	4 (33.3%)
Schizophrenia	354	110 (31.1%)	548	93 (17.0%)
Paraphrenia	256	137 (53.5%)	206	58 (28.2%)
Confusional states	41	29 (70.7%)	165	107 (64.8%)
Organic states****	50	2 (4.0%)	90	8 (8.9%)
Hypochondria	21	13 (61.9%)	0	0
Hysteria	99	70 (70.1%)	29	16 (55.2%)
Anxiety states	308	242 (78.6%)	92	56 (60.9%)
Anxiety hysteria	58	43 (74.1%)	21	13 (61.9%)
Neurasthenia	46	30 (65.2%)	22	12 (54.5%)
Obsessional states	29	18 (62.1%)	13	4 (30.8%)
Psychopathic states	10	4 (40.0%)	11	5 (45.5%)

*Extracted from Reports of Inspector-General of Mental Hospitals^14–18^.

**The terminology and order used for the mental disorders as appears in the reports.

***MDP – Manic Depressive Psychosis.

****demented states, dementia paralytica, congenital mental deficiency, epilepsy, and other states combined due to small numbers.

## Discussion

The main finding of this study is that the clinical indications for the early use of ECT in NSW differed from contemporary practice in NSW 2013 to 2022. Although in both eras ECT was most frequently prescribed for depression, in the 1940s a higher proportion of cases had non-affective psychoses (paraphrenia/schizophrenia – 37.6%) compared with contemporary practice (schizophrenia, schizoaffective and schizophrenia-related disorders – 24.7%), perhaps not surprising at a time when there were no antipsychotic drugs.^20^ In contrast, the sex ratio was almost identical to contemporary practice,^20^ as was the mortality rate,^21^ although it is unclear for what time period after ECT mortality was reported.

The effective use of ECT in anxiety states and other non-psychotic disorders, which might seem unusual to current practitioners, had an evidence base in the 1940s–50s, with good results being reported, including from Australia.^7,14,22^ This was an era where the main treatment for these disorders was psychodynamic psychotherapy, a treatment modality unsuitable for some patients and not accessible for many. It is noteworthy that nearly all the ECT for these conditions was given at Broughton Hall, a hospital predominantly for voluntary patients, and thus with a different patient population to the other mental hospitals.

Nevertheless, psychiatric diagnoses were marred by a lack of reliability and standardisation in the mid-twentieth century. It is possible that some patients with non-melancholic, non-psychotic depressive disorder or agitated depression were misdiagnosed with anxiety states such as hysteria and neurasthenia, which may explain the high response rate for anxiety disorders. ECT remains an indication for non-psychotic depressive disorder.^23^ In addition, having a ‘manic depressive psychosis’ diagnosis did not mean that the patient was psychotic during the episode. Another possibility is that ECT was used for ‘diagnostic-curative purposes’, especially to differentiate between psychogenic and organic disorders in presentations of pain or seizures, and the belief that some patients concealed psychotic symptoms that would be uncovered by ECT, like with the use of pentothal.^14^

That 64.8% of patients with confusional states were reported as having recovered after ECT is surprising given the lack of evidence supporting the efficacy of ECT for delirium.^24^ The most likely reason is that patients with mood or psychotic disorders and altered consciousness or disorientation were described as ‘confused’, which in older patients might be due to a comorbid cognitive disorder. Delirious mania is another example of a diagnostic category with such mixed features.^25^

The use of ECT in other organic disorders including dementia had, not unexpectedly, poor outcomes. While the data does not indicate the reason ECT was used, it is likely that it was used to treat behavioural or psychotic features of these disorders, as was reported for treating severe depression and agitation in dementia paralytica in 1953 and more recently in severe behavioural disturbances in people with dementia.^26,27^

Better outcomes occurred in the affective than non-affective psychoses, as had been reported in studies from the United States in 1945 and 1948.^22,28^ While there was no information about relapse rates, that around half of the patients receiving ECT at Gladesville Hospital in 1948/49 had previous courses of ECT suggests that over time recovery was not sustained. In addition, the reduced effectiveness of ECT in 1948/49 compared with 1944/45 is consistent with a higher proportion of treatments being given to relapses.

Although the proportion of patients who received ECT in each of the seven hospitals is unknown, the marked variation in the number of treatment courses is noteworthy. More than half of all courses were administered in Broughton Hall, a voluntary admission clinic which may have attracted more people of higher socio-economic status, a non-clinical predictor of access to ECT in the current day.^20^

The three hospitals which were least likely to report ECT were the only hospitals outside of Sydney, collectively accounting for 13.6% of all courses, although the Commonwealth Census of 1947 reported 28% of the NSW population was rural dwelling.^29^ Whilst this does suggest a geographical disadvantage for receiving ECT, it may simply have reflected the slower spread of information about a new treatment in that era.

Finally, whilst there is no information about the manner in which treatment was administered in NSW in the 1940s, it would be expected to have been similar to that used in Queensland in the same period.^14^ Apprehensive or restless patients were given a mixture of potassium bromide, chloral hydrate and paraldehyde for sedation during treatment; others received paraldehyde immediately before treatment to minimise post-ictal agitation. In the absence of muscle relaxants, a wardsman firmly pressed down on the patient’s shoulders, one nurse held down the arms and another the jaw to ensure the mouthguard remained stable. For most patients, electrode placement was over the frontal areas and the electrical dose delivered was 140 V over 0.3 seconds. Whilst subsequent research on the use of bilateral electrode placements using ultra-brief pulse-width has been limited, current ECT guidelines have warned that such treatment is of relatively low efficacy.^30^

Some limitations to this study include the lack of information regarding patient age, the number of treatments received in each course, the way ECT was administered, and how many had a previous ECT course.

In conclusion, this study documents the early use of ECT in NSW, the broader range of mental conditions for which it was prescribed compared with contemporary practice, the declining effectiveness of treatments over the 5-year period, and that better outcomes were obtained for affective than non-affective psychoses.
